# Single Coronary Artery as a Rare Developmental Variant in Cardiac Computed Tomography Angiography

**DOI:** 10.3390/diagnostics13081369

**Published:** 2023-04-07

**Authors:** Paweł Gać, Agnieszka Trejtowicz-Sutor, Rafał Poręba

**Affiliations:** 1 Centre for Diagnostic Imaging, 4th Military Hospital, Weigla 5, PL 50-981 Wroclaw, Poland; 2 Department of Population Health, Division of Environmental Health and Occupational Medicine, Wroclaw Medical University, Mikulicza-Radeckiego 7, PL 50-368 Wroclaw, Poland; 3 Department of Internal and Occupational Diseases, Hypertension and Clinical Oncology, Wroclaw Medical University, Borowska 213, PL 50-556 Wroclaw, Poland

**Keywords:** cardiac computed tomography angiography, developmental variant, single coronary artery

## Abstract

Cardiac computed tomography angiography (CCTA) is a non-invasive method for the diagnosis of coronary artery disease. In addition to the assessment of possible stenoses in the coronary arteries, this method also allows the assessment of other abnormalities of coronary and extracoronary heart structures. CCTA is the optimal method for assessing the relationship of coronary arteries to other anatomical structures; thus, it is used as a method of diagnosing developmental variants of coronary circulation. We present images of a single left coronary artery in a 384-slice CCTA in a 69-year-old Caucasian female patient with non-specific chest pain and low intermediate cardiovascular risk as an example of a rare developmental coronary variant. In conclusion, the importance of CCTA as a method of diagnosing developmental variations of the heart and vessels should be emphasized.

We present an image of a rare developmental variant of the coronary circulation on computed tomography angiography (CTA) in the form of a single left coronary artery in a 69-year-old Caucasian female patient with non-specific chest pain and low intermediate cardiovascular risk by clinical assessment, referred from the cardiology clinic to the computed tomography (CT) laboratory to exclude significant coronary artery disease.

Coronary computed tomography angiography (CCTA) was performed using a 384-slice Siemens Somatom Force CT scanner (Siemens Healthineers, Erlangen, Germany). The total coronary artery calcium score was 34.9 (left anterior descending artery calcium score 34.9). The risk of significant coronary artery disease based on the coronary artery calcium score was estimated as mild (Figure 1).

The angiographic phase revealed the presence of a patent left main coronary artery (LM), which divides into three patent arteries: the left anterior descending artery (LAD) giving off the diagonal branch (Dg), the narrow intermediate branch (IR) and the dominant left circumflex artery (LCx) with numerous obtuse marginal branches (OM). The first three obtuse marginal branches (OM1-OM3) were well developed, and the following (OM4-OM7) were narrow. The narrow OM5 is in the posterior interventricular sulcus (replacing the typical posterior interventricular artery PDA). The terminal part of the LCx located in the circumflex sulcus to the right of the posterior interventricular sulcus is “the replaced right coronary artery”. The examination showed no artery branching from the right coronary sinus, i.e., a typical right coronary artery (RCA) (Figure 2).

The presence of calcified atherosclerotic plaque in the proximal segment of the LAD was demonstrated, causing short-section stenosis of the vessel by 25–50% (Figure 3).

No other stenoses in the coronary arteries were observed. The obtained image of lesions in the coronary arteries made it possible to exclude significant coronary artery disease; it corresponded to score “2” in the CAD-RADS classification. Images obtained during the angiographic phase allowed for the identification of a developmental variant of the coronary arteries in the form of a single left coronary artery originating from the left coronary sinus.

The CCTA showed normal left ventricular systolic function (ejection fraction EF: 80%, myocardial mass LVM: 111.78 g, end-diastolic volume EDV: 113.73 mL, end-systolic volume ESV: 22.59 mL, stroke volume SV: 91.14 mL; Figure 4).

Normal aortic and mitral valves were visualized. There was minimal fluid volume in the pericardial sac. A variant of the ostia of the pulmonary veins to the left atrium has been demonstrated: three pulmonary veins on the right side (RSPV—right superior pulmonary vein, RMPV—right middle pulmonary vein (middle lobe vein) and RIPV—right inferior pulmonary vein) and a single pulmonary vein on the left side (LPV; Figure 5).

Anomalies in the structure of the coronary arteries are rare; in the general population, they affect about 1.3% (range 0.3–5.64%) of people [1,2]. Most often, anomalies of the coronary arteries do not cause clinical symptoms. They can be detected incidentally in examinations such as coronary angiography or multislice computed tomography [3]. In symptomatic cases, the first manifestation may include palpitations, angina pectoris, exertional dyspnoea, arrhythmia, myocardial infarction and even sudden cardiac death [2]. The defect in the form of a single coronary artery originating from the aorta and supplying the whole heart is found in an even smaller percentage of the population and occurs in <0.1% of people [4,5] or, according to Lipton et al., in 0.024–0.066% of people [6].

The anomaly in the form of a single coronary artery, despite its frequent asymptomatic course, impairs blood flow through the myocardium. People with this defect have an increased risk of sudden cardiac death, which is explained by the predisposition to the early occurrence of atherosclerosis due to high coronary blood flow [7]. The most common classification used to assess the course of a single coronary artery is the Lipton classification which was later modified by Sharbaugh and White. Depending on the location of the orifice, anatomical distribution and the course of its branches, a single coronary artery can be classified into one of three groups [7,8,9]. In detecting anatomical differences of the coronary arteries, including a single coronary artery, the following factors are important: conventional coronary angiography, computed tomography angiography (CTA) and magnetic resonance angiography (MRA). It has been shown that the images obtained in CTA correlate with conventional coronary angiography [10]. The advantage of CTA is its non-invasiveness, high temporal and spatial resolution, three-dimensional image reconstruction and, additionally, the possibility of excluding co-occurring cardiac dysfunction [11].

In conclusion, the importance of CCTA as a method for recognizing rare developmental changes of the heart and vessels should be emphasized. Due to the excellent spatial resolution, good tissue resolution and the ability to determine the anatomical relationships between the cardiovascular structures and the surrounding extracardiac structures, CCTA is the optimal diagnostic method for this type of developmental changes.

## Figures and Tables

**Figure 1:**
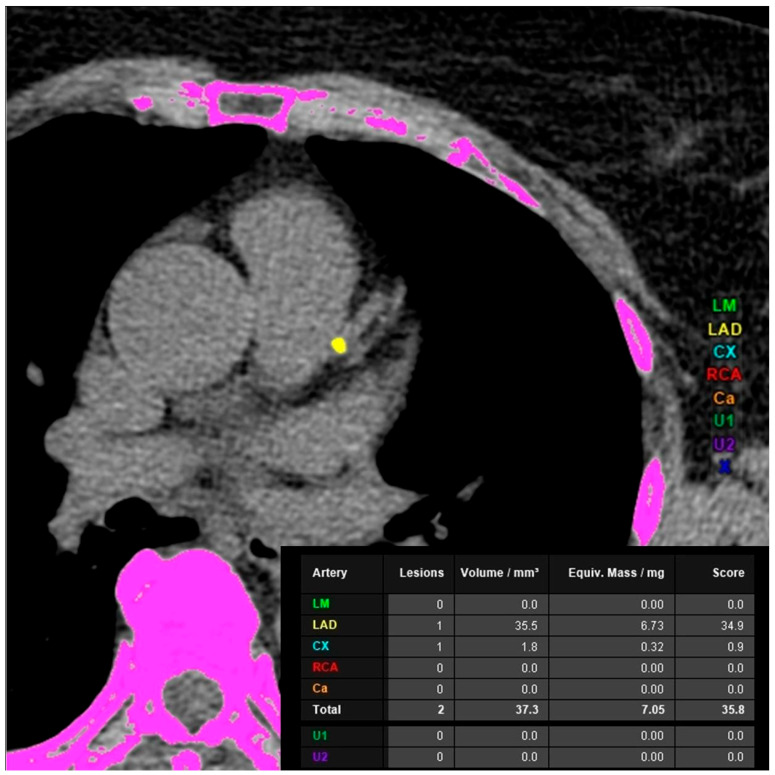
Cardiac computed tomography angiography. Native phase. Coronary artery calcium score assessment—mild risk of significant coronary artery diseases. LM—left main coronary artery, LAD—left anterior descending artery, CX—left circumflex artery, RCA—right coronary artery, Ca—minor branches of the coronary arteries, U—extracoronary calcification.

**Figure 2:**
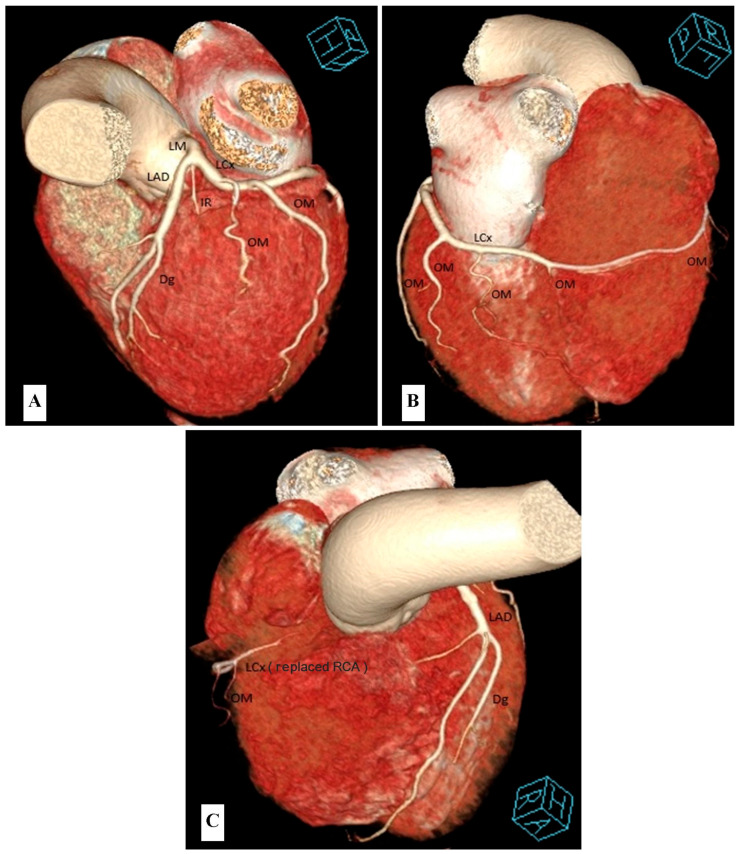
Cardiac computed tomography angiography. Angiographic phase. Volume Rendering Technique (VRT) reconstruction. Coronary circulation—single coronary artery. LM—left main coronary artery, LAD—left anterior descending artery, Dg—diagonal branch, IR—intermediate branch, LCx—left circumflex artery, OM—obtuse marginal branch, RCA—right coronary artery. (**A**) cranial-posterior-left oblique view. (**B**) caudal-posterior-right oblique view. (**C**) cranial-anterior-right oblique view.

**Figure 3:**
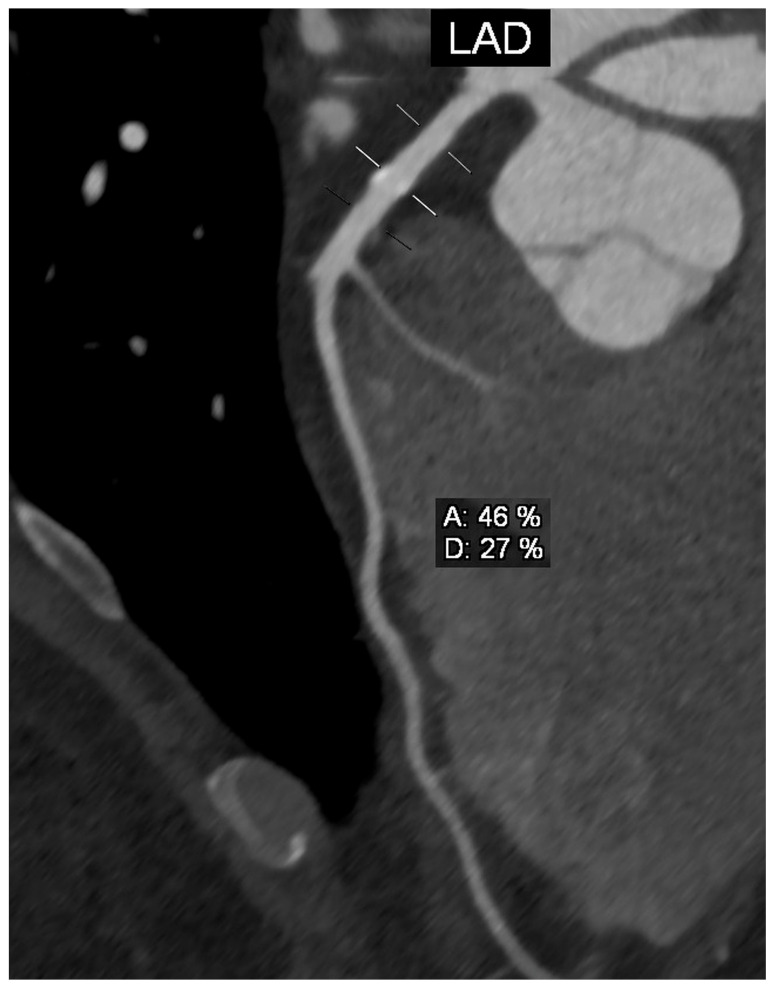
Cardiac computed tomography angiography. Angiographic phase. Curved planar reconstruction (CPR). Left anterior descending (LAD) artery—short-section stenosis of the vessel by 25–50%. Three lines on the course of the vessel: a reference line above the stenosis, a line at the level of the stenosis and a reference line below the stenosis. A—% of stenosis calculated based on the perpendicular cross-sectional area of the vessel, D—% of stenosis calculated based on of the diameter of the vessel.

**Figure 4:**
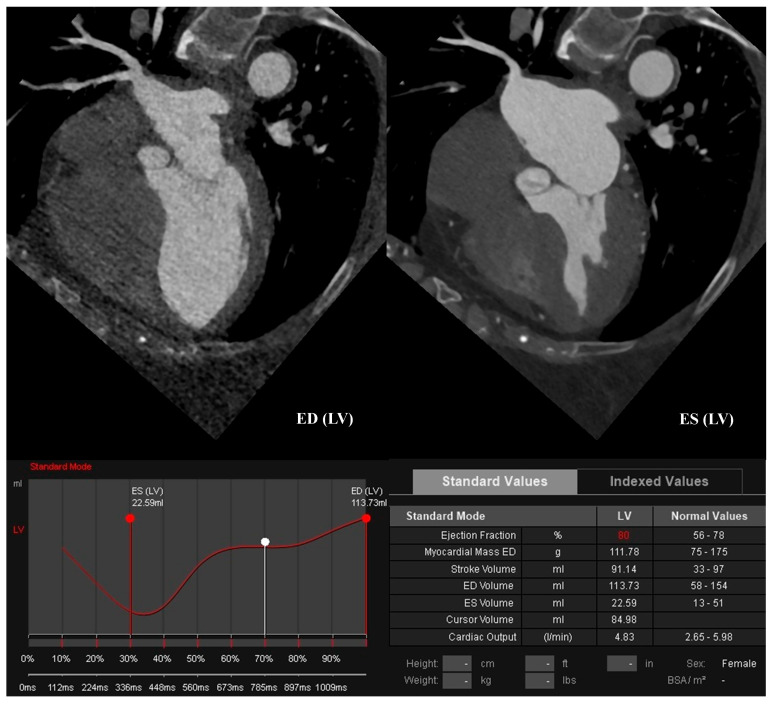
Cardiac computed tomography angiography. Angiographic phase. Left ventricular function assessment—normal left ventricular ejection fraction. ED—end-diastolic, ES—end-systolic, LV—left ventricle.

**Figure 5:**
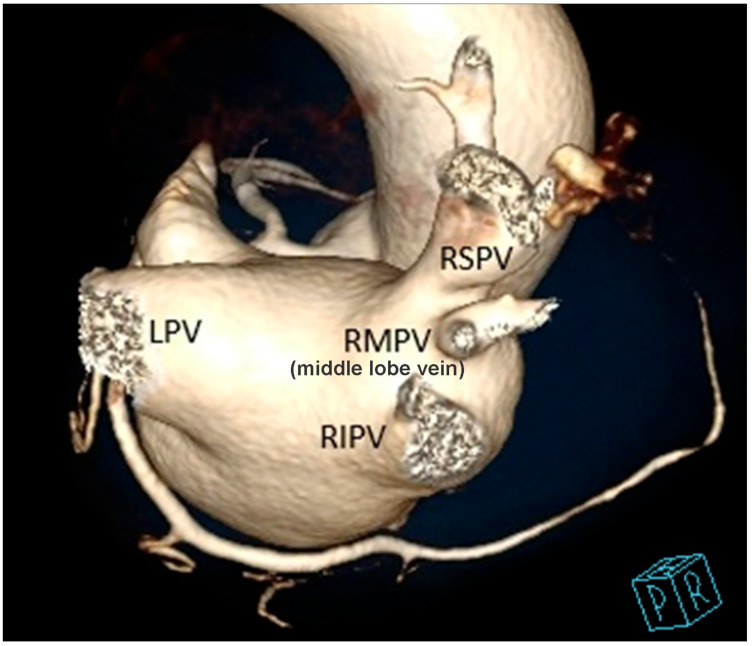
Cardiac computed tomography angiography. Angiographic phase. Volume Rendering Technique (VRT) reconstruction. Cranial-posterior-right oblique view. Pulmonary venous drainage to the left atrium—accessory right middle pulmonary vein. RSPV—right superior pulmonary vein, RMPV—right middle pulmonary vein, RIPV—right inferior pulmonary vein, LPV—left pulmonary vein.

## Data Availability

Not applicable.
